# Quantifying the impact of pesticides on learning and memory in bees

**DOI:** 10.1111/1365-2664.13193

**Published:** 2018-07-10

**Authors:** Harry Siviter, Julia Koricheva, Mark J. F. Brown, Ellouise Leadbeater

**Affiliations:** ^1^ School of Biological Sciences Royal Holloway University of London Surrey UK

**Keywords:** agricultural policy, bees, cognition, insecticide, neonicotinoid, pesticide, pollination, pollinators

## Abstract

1. Most insecticides are insect neurotoxins. Evidence is emerging that sublethal doses of these neurotoxins are affecting the learning and memory of both wild and managed bee colonies, exacerbating the negative effects of pesticide exposure and reducing individual foraging efficiency.
2. Variation in methodologies and interpretation of results across studies has precluded the quantitative evaluation of these impacts that is needed to make recommendations for policy change. It is not clear whether robust effects occur under acute exposure regimes (often argued to be more field‐realistic than the chronic regimes upon which many studies are based), for field‐realistic dosages, and for pesticides other than neonicotinoids.
3. Here we use meta‐analysis to examine the impact of pesticides on bee performance in proboscis extension‐based learning assays, the paradigm most commonly used to assess learning and memory in bees. We draw together 104 (learning) and 167 (memory) estimated effect sizes across a diverse range of studies.
4. We detected significant negative effects of pesticides on learning and memory (i) at field realistic dosages, (ii) under both chronic and acute application, and (iii) for both neonicotinoid and non‐neonicotinoid pesticides groups.
5. We also expose key gaps in the literature that include a critical lack of studies on non‐*Apis* bees, on larval exposure (potentially one of the major exposure routes), and on performance in alternative learning paradigms.
6. *Policy implications*. Procedures for the registration of new pesticides within EU member states now typically require assessment of risks to pollinators if potential target crops are attractive to bees. However, our results provide robust quantitative evidence for subtle, sublethal effects, the consequences of which are unlikely to be detected within small‐scale prelicensing laboratory or field trials, but can be critical when pesticides are used at a landscape scale. Our findings highlight the need for long‐term postlicensing environmental safety monitoring as a requirement within licensing policy for plant protection products.

## 1 INTRODUCTION

A wealth of empirical evidence for global pollinator decline has driven unprecedented interest in the mechanisms by which anthropogenic changes influence both domestic honeybees (*Apis* spp.) and native wild bees (e.g., *Bombus* spp.; Aizen & Harder, 2009; Goulson, Nicholls, Botias, & Rotheray, 2015; Potts et al., 2010). Habitat loss, an increase in the prevalence of bee pathogens, the spread of invasive species, and climate change have all been implicated as potential drivers (Brown & Paxton, 2009; Cameron et al., 2011; Goulson et al., 2015; Kerr et al., 2015; Potts et al., 2010; Winfree, Aguilar, Vázquez, Lebuhn, & Aizen, 2009; Woodcock et al., 2016). Recently, considerable attention has also been devoted to the contribution of agricultural pesticides, and particularly neonicotinoids, which are present in the nectar and pollen of treated crops and nearby wildflowers, and thus in colony food‐stores (Mitchell et al., 2017; Simon‐Delso et al., 2015).

There is strong evidence to associate pesticide use with bee population decline (Woodcock et al., 2016) and consequently with potential losses to pollination services and crop yields (Stanley & Raine, 2016; Stanley, Garratt, et al., 2015). At the colony level, pesticide exposure is associated with negative impacts on fitness‐determining traits that include colony initiation, colony growth, and reproductive output (Arce et al., 2017; Baron, Jansen, Brown, & Raine, 2017; Baron, Raine, & Brown, 2014, 2017; Rundlöf et al., 2015; Tsvetkov et al., 2017; Whitehorn, O'Connor, Wackers, & Goulson, 2012; Woodcock et al., 2017). The mechanisms that underlie these effects remain unclear, but pesticides have been shown to negatively impact key aspects of worker performance including foraging efficiency and navigation ability (Feltham, Park, & Goulson, 2014; Gill & Raine, 2014; Gill, Ramos‐Rodriguez, & Raine, 2012; Henry et al., 2012; Stanley, Russell, Morrison, Rogers, & Raine, 2016). Models of colony growth predict that such small negative impacts on a limited cohort of workers can have severe negative consequences downstream in the colony cycle (Bryden, Gill, Mitton, Raine, & Jansen, 2013).

Many insecticides are neurotoxins that alter synaptic function within the insect central nervous system (Goulson et al., 2015). For example, neonicotinoids and sulfoximines bind to nicotinic acetylcholine receptors (NAChRs), disrupting cholinergic transmission, which can lead to neural cells failing to develop or being inactivated (Palmer et al., 2013; Peng & Yang, 2016), whereas fipronil (a phenylpyrazole) inhibits GABA signalling (El Hassani, Dupuis, Gauthier, & Armengaud, 2009; Moffat et al., 2015, 2016) and can increase neural cell death (Boitard, Devaud, Isabel, & Giurfa, 2015). The mushroom bodies are a neural region specifically associated with olfactory learning and memory in bees (Devaud et al., 2015; Hourcade, Muenz, Sandoz, Rossler, & Devaud, 2010), and there is now strong evidence that mushroom body development and function can be directly impaired through chronic or acute exposure to NAChR agonists respectively (Palmer et al., 2013; Peng & Yang, 2016). The potential consequences for learning and memory are of concern because cognitive abilities are integral to bee foraging. Bees are one of the few taxonomic groups in which there is empirical evidence that directly links cognitive abilities with foraging efficiency, a fitness‐determining trait (Raine & Chittka, 2008). The nectar and pollen rewards offered by floral resources change over time (Heinrich, 2004), and individuals must not only remember which flower species are currently rewarding, but also their location, how to handle different flower types, which inflorescences have just been visited, and where the nest is located (Chittka & Thomson, 2001; Gegear & Laverty, 2001; Heinrich, 2004). Consequently, numerous studies have set out to examine the effects of pesticides on cognitive traits (Klein, Cabirol, Devaud, Barron, & Lihoreau, 2017).

Narrative reviews have highlighted the challenge of drawing general conclusions about pesticide impacts on bees (Godfray et al., 2014, 2015; Goulson et al., 2015; Wood & Goulson, 2017). This is largely due to considerable variation in methodologies. Pesticide dosage, for instance, varies across experiments, as does the definition of a field‐realistic dose (Godfray et al., 2014). Studies also follow alternative exposure‐regime strategies in attempts to mimic field realistic scenarios. While foraging bees may be acutely exposed through consumption during one foraging bout, chronic exposure may occur through repeated foraging on a large pesticide‐treated food source that flowers over a prolonged period, such as oil seed rape, and may be extended by the presence of pesticides within honey and pollen stores (Mitchell et al., 2017). Impacts might also vary across bee genera. For instance, some evidence now suggests that pesticides could differentially affect honeybees (*Apis*) and bumblebees (*Bombus*), with honeybees appearing to be more vulnerable to pesticides in relation to their cognitive abilities than bumblebees under some circumstances (Piiroinen & Goulson, 2016). Finally, effects of pesticides on bee cognition may vary across classes of pesticides, reflecting different modes of action (Klein et al., 2017). Such variation is important as certain neonicotinoids (imidacloprid, clothianidin, and thiamethoxam) are now under a total ban in the EU with respect to agricultural use outside of permanent greenhouse structures (to be implemented by December 2018) (European Commission 2018), which is likely to create market demand for other pesticides as replacements (Brown et al., 2016; Campbell, 2013).

Sublethal effects are more difficult to detect than direct effects on pollinator mortality in small‐scale field laboratory and field trials, but may have critical impacts on pollinator health at the landscape scale. There is thus an urgent need to synthesize the literature assessing sub‐lethal effects in order to provide robust evidence‐based conclusions for policy makers. Here, we quantitatively explore the evidence for sub‐lethal effects of pesticides on bee cognition through meta‐analysis. This enables us to measure the magnitude of the effects of pesticides on bee learning and memory, to explore the sources of heterogeneity underlying these effects (Koricheva, Gurevitch, & Mengersen, 2013), and to identify evidence gaps in the current literature. Specifically, our analysis aimed to answer five questions:

1. Do pesticides negatively affect the learning ability and memory of bees?
2. Do field realistic dosages of pesticides significantly affect bee learning and memory?
3. Do chronic and acute exposure differentially affect learning and memory?
4. Are honeybees and bumblebees differentially affected by pesticides?
5. Do neonicotinoids affect bee learning and memory more than other pesticides?

## 2 MATERIALS AND METHODS

### 2.1 Scope and search strategy

We focused upon olfactory learning and memory, which are typically assessed in bees through an olfactory proboscis extension reflex paradigm (hereafter PER). During a PER experiment, a harnessed bee learns to associate a previously unrewarded scent with sucrose. Bees initially exhibit proboscis extension as an unconditioned response (UR) to antennal contact with sucrose (the unconditioned stimulus; US). When this contact is paired with a scent (the conditioned stimulus; CS), the bee learns to extend its proboscis in response to the scent alone (a conditioned response; CR). Typically, PER‐based experiments that relate to pesticides use an absolute conditioning paradigm (where bees learn to associate only one scent with sucrose) rather than differential conditioning (where one scent is rewarded and an alternative is not; Stanley, Smith, & Raine, 2015). Although other paradigms to test learning and memory (e.g., free‐flying association, spatial learning, aversive learning, or tactile learning [Bernadou, Démares, Couret‐Fauvel, Sandoz, & Gauthier, 2009; Tan et al., 2014; Samuelson, Chen‐Wishart, Gill, & Leadbeater, 2016; Zhang & Nieh, 2015]) are available and widely used in the cognitive literature, only a very small number of studies have used such methods to assay how pesticides influence performance (see Section 4; Bernadou et al., 2009; Samuelson et al., 2016; Zhang & Nieh, 2015). In contrast, the PER paradigm is the most commonly used methodology to assess bee learning and memory and thus provides an obvious target for our study.

We used Web of Science and Google Scholar as search databases (search performed in April 2018). The search criteria used in Web of Science were (“pesticide*” OR “insecticide*” OR “neonicotinoid*”) AND (“bumblebee*” OR “bumble bee*” OR “honey bee*” OR “honeybee*” OR “bee*” OR “*apis*” OR “*bombus*”) AND (“learning” OR “memory” OR “PER” OR “cognition” OR “proboscis extension reflex” OR “proboscis extension response”). After the Web of Science search we used the same key words in Google Scholar and checked the first 200 results, which yielded three additional papers (Figure S1). Twenty‐three papers remained eligible after title and abstract screening, and applying inclusion criteria (see below and Table S1). All 23 papers had their reference lists examined and we did not find any additional data.

### 2.2 Inclusion criteria, data extraction, and final database

To be included in the meta‐analysis, a study had to involve oral exposure of bees to a pesticide followed by an assay of learning and/or memory via a PER conditioning paradigm. Studies were excluded if they did not contain a control group (no pesticide exposure) or if we were unable to extract the means, the standard deviations, and the sample sizes for both the control and the treatment groups. Some raw data were available online (*N* = 3), but in most cases (*N* = 17) the means and standard deviations could be extracted from graphs using WebPlotDigitizer (https://automeris.io/WebPlotDigitizer/). In cases where information was not available, some authors were successfully contacted (*N* = 3). We excluded experimental groups where the bees had been exposed to multiple stressors (e.g., both parasites and pesticides), as we could not be sure which stressor was potentially causing an effect. In all studies included in the analysis, bees were tested either directly or 24 h after pesticide exposure. We excluded one study where the postexposure testing period varied (with delays of up to 11 months; Table S1). After sensitivity analysis (see below) the 23 papers included in the final database (see Table S1) yielded 104 effect sizes for the influence of pesticides on learning ability from 23 papers and 167 effect sizes from 19 papers for the influence of pesticides on memory. These studies were published between 2009 and 2017.

PER experiments use varying criteria to assess learning performance, including the number of trials in which the bee responded to the CS, the first trial in which it responded, or mean performance in a specified batch of trials. For example, Stanley, Smith, et al. (2015) used 15 learning trials (trials in which the UR and the CS are paired) per condition, whereas Piiroinen, Botías, Nicholls, and Goulson (2016) tested their bees over 10 trials. To enable direct comparison, we redefined learning across studies as the proportion of bees that responded positively to the CS by the final learning trial (intertrial interval; mean = 8.17 ± 5.6). Similarly, we collated memory data (the number of bees responding to the CS) from all reported time lengths (range: 10 min–48 h) into two categories that approximate short‐ and long‐term memory (see below). Note that these timings reflect neurologically distinct processes in bees, the transition from short‐ to long‐term memory being translation‐dependent (reviewed in Menzel, 2012).

### 2.3 Potential moderators

Moderators are used in meta‐analysis to investigate the sources of variation in effect sizes between studies (Koricheva et al., 2013). Our meta‐analysis included the following as potential moderators of the size of the effect that pesticide exposure had on learning and memory: pesticide exposure regime (chronic or acute), dosage (field realistic or above), pesticide type (neonicotinoid or other), and genus (*Apis* or *Bombus*). For the memory data, we also included short (<24 h) and long‐term (≥24 h) memory retention as a potential moderator (see below for full models). The treatment was considered acute when the bees were exposed to one dosage of pesticide and chronic when the bees were repeatedly exposed over a sustained period of time, which varied between experiments from 4 days (Williamson & Wright, 2013; Yang, Chang, Wu, & Chen, 2012) to 24 days (Stanley, Smith, et al., 2015).

The definition of a field‐realistic dose is highly contentious and the toxicity of different pesticides varies. To standardise this, we categorized dosages as field‐realistic or above based on pesticide concentrations in nectar, pollen, honey, and bee‐bread extracted from the following sources: Bonmatin et al. (2015), Glaberman and White (2014), Sanchez‐Bayo and Goka (2014). Where more than one estimate was available for a given pesticide we took the mean value (see Table S2 for individual pesticides). For the acute dosages, the nectar pesticide concentration data were further combined with the mean amount of nectar that bees are able to ingest in one foraging bout (40 ng for honeybees; 37.7 ng for bumblebees; Table S3) to calculate the field realistic dose (Cresswell, 2011; Samuelson et al., 2016). Dosages higher than the above thresholds were considered not field realistic.

### 2.4 Meta‐analysis

All analyses were conducted in R (version 1.0.136) using the package *metafor* (Viechtbauer, 2010). Data for learning and memory were analysed separately. We used standardized mean difference in bee learning ability or memory between the control groups and the treatment groups (Hedges’ d) as a measure of effect size (calculated using “escalc” function in *metafor*). For both datasets, we fitted random effects models to calculate the grand mean effect as well as the group means (e.g., effects of acute vs. chronic exposure). The restricted maximum likelihood approach (REML) was used to estimate the parameters of the meta‐analysis models. For each of the two datasets, meta‐regression was then used to explore the sources of variation in effect sizes by including all the moderators (see above) within a single model. Pesticide type was not included in these models because a subset of studies simultaneously exposed bees to more than one pesticide (Williamson, Baker, & Wright, 2013; Williamson & Wright, 2013), which would have led to these studies being dropped from the analyses (for full list of pesticides in meta‐analysis see Table S2). Consequently, we analysed pesticide‐type in a submodel that excluded these studies. “Study” was included as a random factor in all the models to control for potential nonindependence of multiple effect sizes from the same study.

We initially included in the analysis results from studies where bees were exposed to pesticides as larvae. However, there were very few of these (three studies for learning data and two studies for the memory data) and we found that the overall effect of pesticides on bee learning when these studies were included in the overall analysis was much stronger (*d* = −0.60, 95% CI = −0.90 to −0.30), whereas the overall effect of pesticides on bee memory was similar (*d* = −0.24, 95% CI = −0.28 to −0.20) compared to the effects based on the analysis when larval data were excluded from the analysis (see Section 3 for comparison). Thus, to preclude bias, we removed these studies from subsequent analyses. Furthermore, given the small number of studies conducted on bumblebees compared to honeybees, we conducted sensitivity analysis with studies that used honeybees only (see Figure S2). Within this analysis we also compared the impact of pesticides between the European (*Apis mellifera)* and the Asian honeybee (*Apis cerana*) (see Figure S2). We also re‐ran the overall analysis without studies that used multiple pesticides (learning *n* = 2 and memory *n* = 2) and the results did not change (see supplementary material). We tested whether the number of learning trials undergone by the bees influenced the results and found no significant effect (*p* = 0.15) and thus we did not include this factor in the overall model. To test for any potential publication bias, a trim‐and ‐fill technique was used on both the learning and memory data (Duval & Tweedie, 2000).

## 3 RESULTS

**Figure 1:**
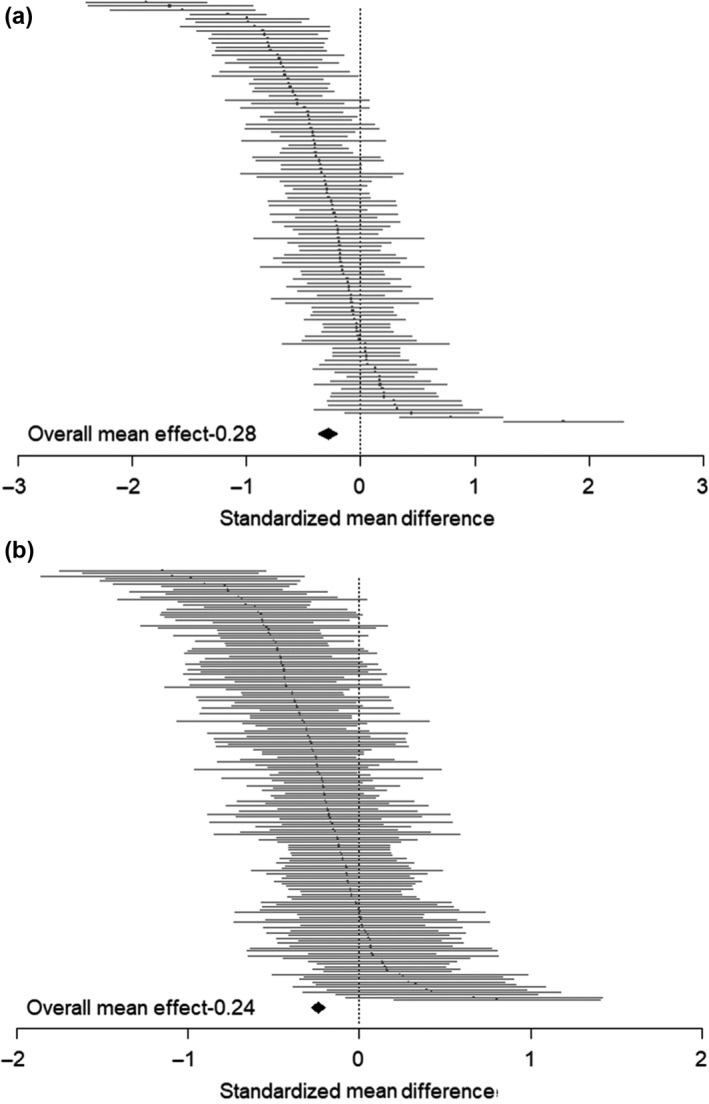
Hedges’ *d* values ± 95% confidence intervals for effects of pesticides on (a) learning ability (b) memory

Overall, pesticide exposure had a significant negative effect on both learning score (*d* = −0.28, 95% CI = −0.36 to −0.20; Figure 1a) and memory (*d* = −0.24, 95% CI = −0.28 to −0.20; Figure 1b). The proportion of between‐study heterogeneity for the learning data was high (*I* ^2^ = 75.61%) but lower for the memory data (*I* ^2^ = 31.51%). When mean effects were recalculated after adjusting for a possible publication bias with a trim‐and‐fill technique, the effect size estimates did not change for the learning results (*d* = −0.28, 95% CI = −0.36 to −0.20; Figure S3) and also showed no bias for the memory data (*d* = −0.28, 95% CI = −0.32 to −0.24; Figure S4).

**Figure 2:**
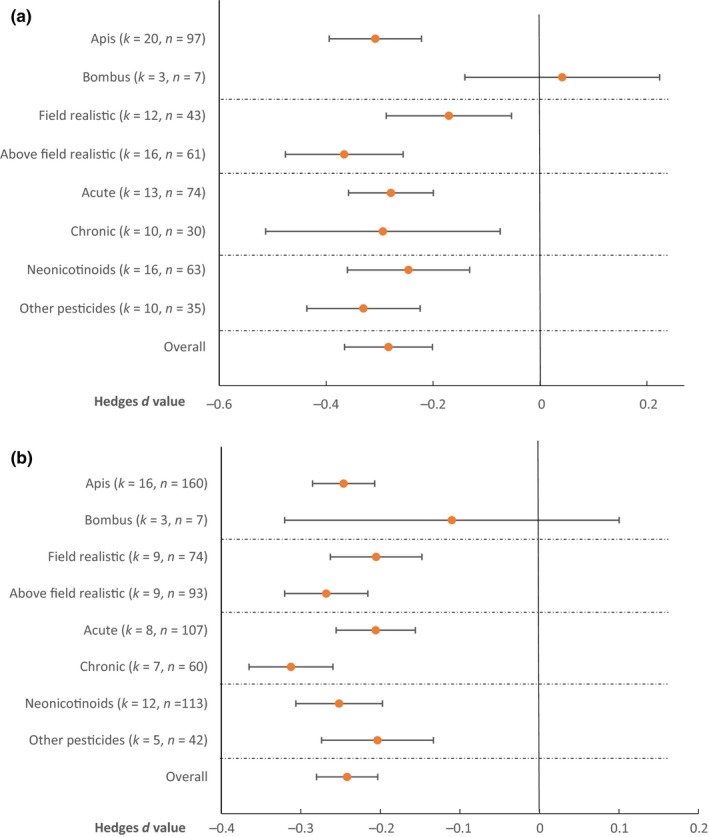
Mean effect size estimates (± 95% confidence intervals) for subsets of the data on the effects of pesticides on (a) learning and (b) memory. Number of studies (*k*) and number of effect sizes (*n*) are given for each subgroup [Colour figure can be viewed at wileyonlinelibrary.com]

While both field realistic and higher doses of pesticide had significant negative effects on learning and on memory, as expected, effects were significantly larger at higher doses (*p* < 0.05 in both cases; Figure 2a,b). Both chronic and acute pesticide exposure had significant negative effects on learning score (Figure 2a), with no significant difference between their effects (*p* = 0.08). In contrast, chronic exposure had a significantly stronger negative impact than acute exposure on memory (*p* < 0.05, Figure 2b). We also found that learning scores of honeybees were more negatively affected by pesticides than those of bumblebees (*p* < 0.05), but these results need to be interpreted with caution given that the majority of studies focused on honeybees. In contrast, while the same trend was present for the effects of pesticides on memory, there was no significant difference between bee species (*p* > 0.05). We found no difference between the effects of neonicotinoids and other pesticides on learning score (*p* = 0.29) or on memory (*p* = 0.14). Finally, there were no differences between effects of pesticides on long‐term (24 h and longer) and short‐term (less than 24 h) memory retention (*p* = 0.47).

## 4 DISCUSSION

Our findings draw together a body of evidence to produce quantitative estimates of the magnitude of pesticide effects on bee learning and memory, across a range of dosage regimes and pesticide treatments. Importantly, our results confirm that pesticide exposure has a significant negative impact on bee learning and memory at field‐realistic doses. Chronic pesticide exposure had a stronger effect on bee memory than acute exposure, although the same effect was not found in relation to learning score. Despite their different modes of action, there were no detectable differences between neonicotinoids and other insecticides in their impacts on learning and memory.

Narrative reviews of pesticide impacts on bees have struggled to draw general conclusions, highlighting the need for a meta‐analytical approach (Godfray et al., 2014, 2015; Goulson et al., 2015; Wood & Goulson, 2017). This tool is particularly valuable when studies show a range of significant and nonsignificant effects. Meta‐analytic assessments of the effects of pesticides on bee biology are currently limited to an analysis of the LD50 paradigm (Arena & Sgolastra, 2014), or a focus on individual pesticides and a specific species (Cresswell, 2011), while one recent meta‐analysis showed that neonicotinoids have a negative impact on performance of beneficial arthropods (Main, Webb, Goyne, & Mengel, 2018). This study provides a significant step forward in our understanding of pesticide impacts on learning and memory, and as such makes progress towards resolving a number of issues in this field.

Firstly, pesticide research has been criticized on the basis that experimental dosages are not field‐realistic (Campbell, 2013; Carreck & Ratnieks, 2014; Godfray et al., 2014, 2015). Here we systematically re‐classified studies based on up‐to‐date estimates of field‐realistic exposure and found significant negative impacts of field‐realistic pesticide doses on learning and memory. Secondly, it has been suggested that chronic pesticide exposure is unrealistic, because wild flowers offer an alternative to pesticide treated crops (Garbuzov, Couvillon, Schürch, & Ratnieks, 2015; Godfray et al., 2014, 2015). Here we have shown that even short‐term (acute) exposure during one foraging bout can significantly impair learning and memory in bees. Chronic exposure had a stronger effect than acute exposure for the memory dataset, potentially because bodily pesticide residues from acute doses may be more likely to have been metabolized before the memory trial than chronic doses, but both chronic and acute doses significantly impaired both learning and memory. Chronic pesticide exposure is increasingly likely to occur in the field as water‐soluble systemic pesticides have been found to occur in wild flowers on field margins (Botias et al., 2015), and in flowers sold in garden centres (Lentola et al., 2017), while pesticide products are freely available for gardeners to purchase, and bees preferentially feed on sucrose solutions that have been treated with pesticides (Kessler et al., 2015). Our results draw together a body of evidence that in combination suggests the rising prevalence of pesticides in the environment (Mitchell et al., 2017) is increasingly likely to influence the cognitive abilities of bees.

The studies used in the analysis assayed the effects of pesticides on learning and memory in adult bees. Pesticides are regularly found in the honey and pollen stores of honeybees, with a recent global study finding neonicotinoids in 75% of all honey samples (Mitchell et al., 2017). Consequently, bee larvae are likely to be exposed to pesticides while developing. Such larvae can take longer to develop, and adult bees show reduced longevity (Wu, Anelli, & Sheppard, 2011). Prior to the removal of larval‐exposure experiments, our results showed a stronger effect of pesticides on bee learning, making our current estimates conservative. This suggests that bees could be more sensitive to pesticide exposure when exposed as larvae. Given that the impacts of larval exposure are relatively unexplored (Peng & Yang, 2016; Tan, Wang, Dong, Li, & Nieh, 2017; Tan et al., 2015; Yang et al., 2012), future research should test whether exposure of bee larvae to field realistic levels of pesticides has a stronger effect on the cognitive abilities of bees than exposure of adults, which could subsequently lead to stronger sublethal effects in the field (Klein et al., 2017).

Our systematic search highlighted a knowledge gap that results from a heavy focus on *Apis*, with a dearth of studies on bumblebees and other wild bees. We found no evidence for an effect of pesticide exposure on bumblebee cognition, but the small dataset available for *Bombus* lacks power, and should be interpreted with caution. There is evidence to suggest that feeding rates drop following pesticide exposure in *Bombus* but not *Apis* (Cresswell, Robert, Florance, & Smirnoff, 2014) which could lead to reduced exposure for *Bombus* over the longer term in chronic experiments. However, the same study found that metabolic breakdown of pesticides was quicker in *Apis* than *Bombus*, with bumblebees maintaining much higher bodily residues than honeybees that were fed the same dose (Cresswell et al., 2014). It is also possible that robust differences exist in target‐site sensitivity, as have been reported in other insects (Lind, Clough, Reynolds, & Earley, 1998; Liu et al., 2005), but such effects are yet to be investigated in *Bombus* and *Apis*. It is too early to draw conclusions about species differences in the impact of pesticides on bee cognitive abilities, and this knowledge gap is important given that wild bee flower visits can enhance the fruit set of crops regardless of the presence of honeybees (Garibaldi et al., 2013), and are thought to offer an important buffer in the case of a domesticated honeybee collapse (Greenleaf & Kremen, 2006). Research on non‐*Apis* species, such as bumblebees (including species other than *Bombus terrestris*) and solitary bees, is sorely needed, and the development of non PER‐based paradigms for testing the effects of pesticides on cognition is welcome in this respect (Samuelson et al., 2016; Tan et al., 2014).

The results also provide no support for differential effects of neonicotinoids in comparison to other pesticides, on bee learning and memory. Neonicotinoids have been a particularly controversial pesticide group because they are typically applied as a seed treatment, resulting in contamination of the pollen and nectar of exposed plants, which are then consumed by bees (Bonmatin et al., 2015). Despite restrictions on their use within Europe, neonicotinoids are the most widely used type of insecticide worldwide (Simon‐Delso et al., 2015), which has driven an abundance of pesticide research focussing on their use. Currently, however, there is not enough available data on other, non‐neonicotinoid pesticide groups (pyrethroids, phosphorothioates, etc.) to make more specific comparisons between effects of neonicotinoids and other classes of neurotoxins. One possible consequence of the European moratorium, and now the total ban of certain neonicotinoids, is the creation of a gap in the market for alternative products to achieve the same effect (Campbell, 2013; Klatt, Rundlöf, & Smith, 2016). Thus, in order for policy makers to make conclusive comparisons between neonicotinoids and other pesticides, future research should focus on generating more data on how other pesticides, including novel pesticides such as sulfoximines (Brown et al., 2016), influence bee cognition.

One limiting factor in the literature to date is that almost all the available data collected so far has derived from a PER paradigm. This paradigm is extraordinarily useful in providing a sensitive means to exclude confounding variables and experimental noise, but several alternative methodologies are available that potentially mimic an ecologically realistic scenario more closely (e.g., Samuelson et al., 2016) as they involve free‐flying bees. Such paradigms may lend themselves more fruitfully to non‐*Apis* species than is the case for PER. Furthermore, pesticide exposure has been shown to influence olfactory processing (Andrione, Vallortigara, Antolini, & Haase, 2016) suggesting that exploration of alternative visual and/or spatial modalities will be critical if researchers are specifically interested in how pesticides influence bee learning and memory at the level of neural processing, rather than stimulus perception. Initial exploration of these methodologies has provided evidence for cognitive effects of pesticides outside of olfactory paradigms, and should be further explored (Samuelson et al., 2016).

A final, and important, knowledge gap that remains is quantification of the link between worker cognitive performance and fitness. Detecting long‐term colony‐level consequences of sublethal stress on pollinators is time‐ and resource‐intensive. In contrast, PER is quick, repeatable, widely used and accessible on a large scale. As such, it could provide a valuable addition to current LD50 methodologies to test effects of pesticides on bees (OECD, 2017). However, linking cognitive traits with fitness measures, such as foraging success, is a major outstanding challenge in the literature (Rowe & Healy, 2014), because it is difficult to control for confounding variables when assaying cognition in a natural environment. However, as central‐place foragers, bee colonies lend themselves to laboratory‐based cognitive testing followed by fitness assays in the wild. Using this type of methodology, bumblebee colony foraging intake has been shown to increase with the proportion of fast learning‐workers (Raine & Chittka, 2008), although more recent research failed to find the same relationship at an individual level (Evans, Smith, & Raine, 2017). Conversely, there is evidence to suggest that bees that are poor learners come across novel resources more frequently, potentially increasing foraging performance (Burns, 2005; Evans & Raine, 2014). The relationship between investment in cognitive performance and colony foraging success is likely to be multifaceted, and is a clear avenue for further exploration.

## 5 CONCLUSIONS

Current interest in the effects of pesticides on pollinators is based upon the need to understand the nature of negative effects in order that they can be reduced via policy change. To this end, the results of this meta‐analysis provide the evidence that pesticides have a significant negative influence on the learning and memory of bees at field realistic exposure levels, confirming that classical ecotoxicological tests are failing to assess the sub‐lethal consequences of pesticide exposure. Our results also highlight evidence gaps that should be addressed in order to move forward. Future research needs to focus on (a) testing how larval pesticide exposure influences cognition, (b) understanding how pesticides influence non‐*Apis* bee species, and (c) generating data on how potential replacements for neonicotinoid pesticides influence bee cognition.

This study demonstrates that meta‐analyses can be used to quantify how pesticides influence bee biology, an approach that could ultimately aid in pollinator conservation. In recognition of the fact that pesticide exposure poses potential risks to pollinators, plant protection product licensing protocols often require evidence of risk assessment to be included with application dossiers. While these policies may promote detection of direct mortality risks, they are unlikely to uncover subtle sublethal effects (such as those demonstrated here) that may have major environmental consequences when pesticides are applied at the landscape scale postlicensing. Our findings thus highlight the need for policies promoting postlicensing environmental safety monitoring for plant protection products, mirroring that which is in place for pharmaceutical products and food safety (Milner & Boyd, 2017).

## AUTHORS’ CONTRIBUTIONS

H.S., E.L., and M.J.F.B. conceived the study. All authors contributed to the data collection design. H.S. collected the data and H.S. and J.K. conducted the statistical analysis. H.S. wrote the original manuscript and all authors contributed to and approved the final version.

## Data Availability

Data available via the Dryad Digital Repository https://doi.org/10.5061/dryad.b2t08b6 (Siviter, Koricheva, Brown, & Leadbeater, 2018).
