# Supplementary figures and images for: Quantifying the impact of pesticides on learning and memory in bees
Source: J Appl Ecol. 2018 Jul 10;55(6):2812–21. doi: 10.1111/1365-2664.13193 (PMC6221055; doi:10.1111/1365-2664.13193)

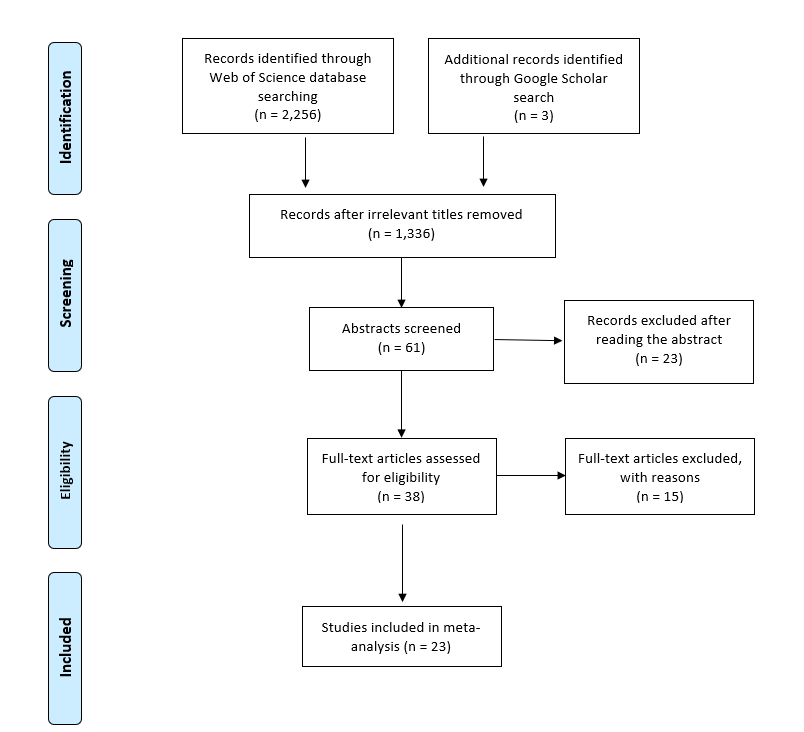

Supplement: Supplementary file 1 [file JPE-55-2812-s001.png]

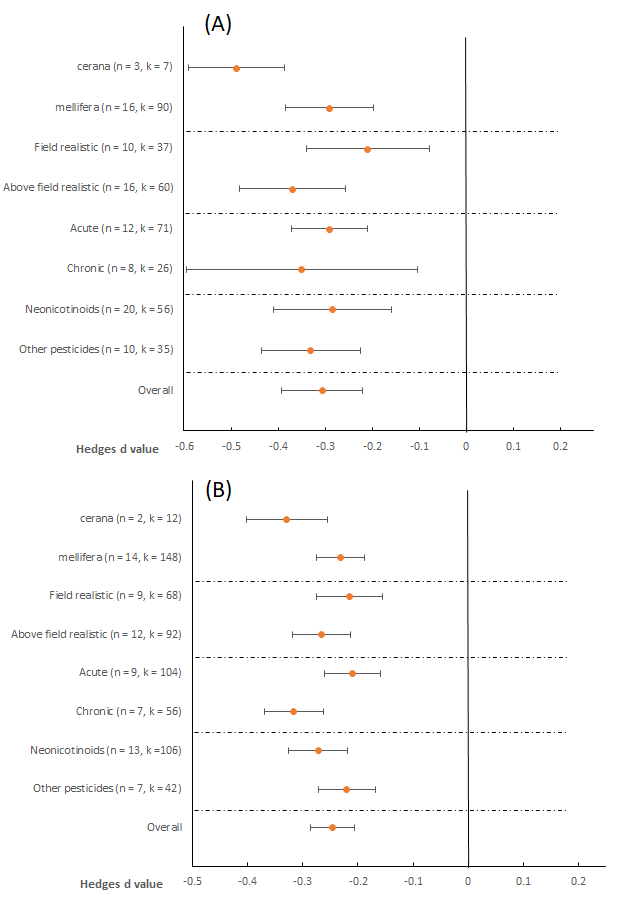

Supplement: Supplementary file 2 [file JPE-55-2812-s002.png]

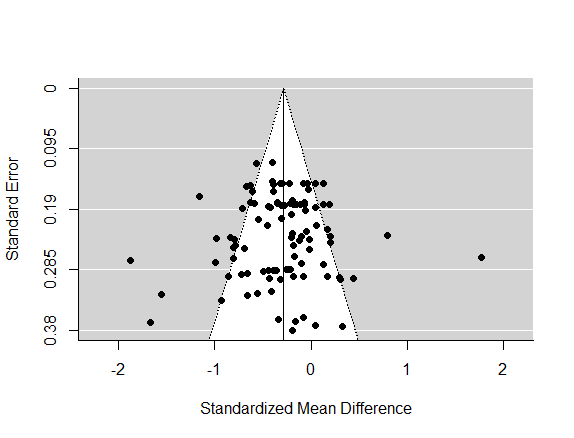

Supplement: Supplementary file 3 [file JPE-55-2812-s003.png]

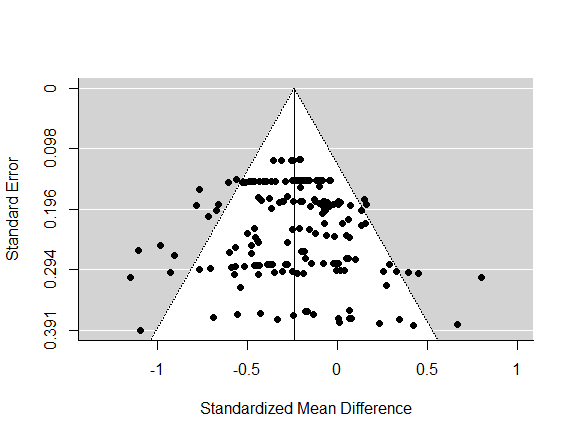

Supplement: Supplementary file 4 [file JPE-55-2812-s004.png]
