# Supplementary material for: Quantifying the impact of pesticides on learning and memory in bees
Source: J Appl Ecol. 2018 Jul 10;55(6):2812–21. doi: 10.1111/1365-2664.13193 (PMC6221055; doi:10.1111/1365-2664.13193)
Supplement: Supplementary file 5 [file JPE-55-2812-s005.docx]

**Quantifying the impact of pesticides on learning and memory in bees**

Harry Siviter, Julia Koricheva, Mark J F Brown, Ellouise Leadbeater

School of Biological Sciences, Royal Holloway University of London, Egham, Surrey, TW20 0EX, UK

**Supplementary material**

**Figure S1: Modified PRISMA flowchart.**


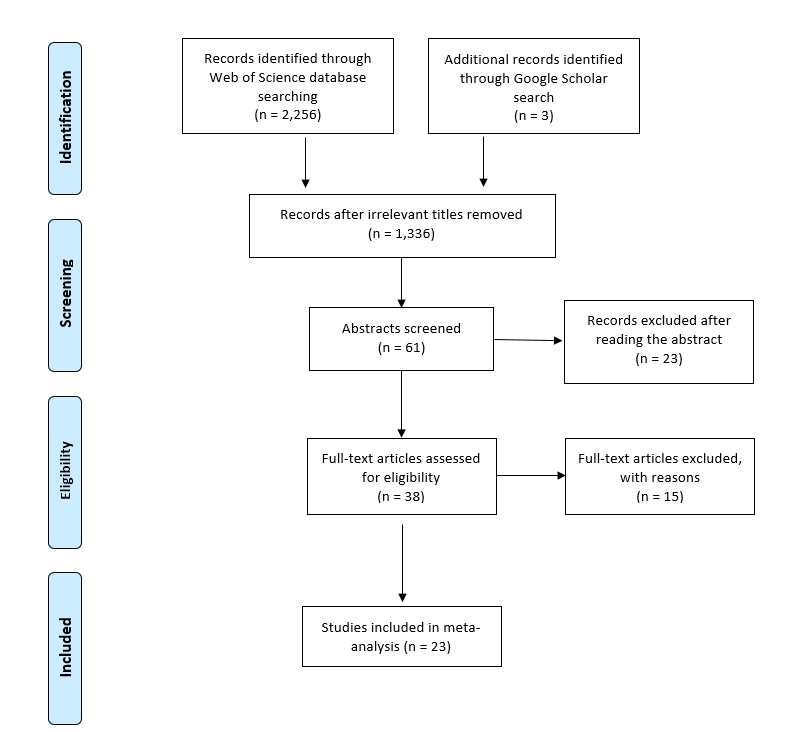


**Table S1. Full list of the papers and reasons why some were not included.**

| Study number | Reference | Included | Reason for exclusion |
| --- | --- | --- | --- |
| 1) | (Alkassab & Kirchner 2016) | ✓ |  |
| 2) | (Piiroinen *et al.* 2016) | ✓ |  |
| 3) | (Urlacher *et al.* 2016) | ✓ |  |
| 4) | (Stanley, Smith & Raine 2015) | ✓ |  |
| 5) | (Goñalons & Farina 2015) | ✓ |  |
| 6) | (Wright, Softley & Earnshaw 2015) | ✓ |  |
| 7) | (Chakrabarti *et al.* 2015) | X | N values not specific and acquisition results not reported |
| 8) | (Tan *et al.* 2015) | ✓ |  |
| 9) | (Frost, Shutler & Hillier 2013) | ✓ |  |
| 10) | (Tan *et al.* 2013) | ✓ |  |
| 11) | (Williamson, Baker & Wright 2013) | ✓ |  |
| 12) | (Williamson & Wright 2013) | ✓ |  |
| 13) | (Yang *et al.* 2012) | ✓ |  |
| 14) | (Ciarlo *et al.* 2012) | ✓ |  |
| 15) | (Schneider, Eisenhardt & Rademacher 2012) | X | Not a pesticide |
| 16) | (Han *et al.* 2010) | ✓ |  |
| 17) | (Aliouane *et al.* 2009) | ✓ |  |
| 18) | (Ramirez-Romero *et al.* 2008) | X | No N values |
| 19) | (El Hassani *et al.* 2008) | ✓ |  |
| 20) | (Ramirez-Romero, Chaufaux & Pham-Delegue 2005) | X | Results for effects of pesticides on learning are not clear |
| 21) | (Decourtye *et al.* 2005) | X | Not clear which n values go with which treatment group |
| 22) | (Abramson *et al.* 2004) | X | N values are not clear |
| 23) | (Decourtye *et al.* 2004) | X | N values are not clear and no SD |
| 24) | (Decourtye, Lacassie & Pham-Delègue 2003) | X | N value are a range – not specific |
| 25) | (Weick & Thorn 2002) | X | Injection not oral |
| 26) | (Decourtye *et al.* 2001) | X | Could not gain access |
| 27) | (Abramson *et al.* 1999) | X | No SD |
| 28) | (Burden *et al.* 2016) | X | Metal not pesticides |
| 29) | (Piiroinen & Goulson 2016) | ✓ |  |
| 30) | (Thany *et al.* 2015) | ✓ |  |
| 31) | (Abramson *et al.* 2012) | ✓ |  |
| 32 | (Tan *et al.* 2017) | ✓ |  |
| 33 | (Tison *et al.* 2017) | ✓ |  |
| 34 | (Papach *et al.* 2017) | ✓ |  |
| 35 | (Hesselbach & Scheiner 2018) | ✓ |  |
| 36 | (Li *et al.* 2017) | X | N values given as a range |
| 37 | (Bonnafe *et al.* 2017) | X | Bees tested 11 months after pesticide exposure or up to 21 days after. Not comparable to the other studies |
| 38 | (Rix & Christopher Cutler 2017) | X | Topically exposed not orally |

**Table S2. The average and maximum concentrations of pesticides found in the pollen and nectar of crops and plants and in the nectar and pollen content found in bee colonies (Glaberman & White 2014; Sanchez-Bayo & Goka 2014; Bonmatin *et al.* 2015)**

| Pesticide | Pesticide Type | Average (ppb) | Maximum (ppb) | Reference |
| --- | --- | --- | --- | --- |
| Acetamiprid | Neonicotinoid | 12.266 | 112.8 | (Sanchez-Bayo & Goka 2014) |
| Chlorpyrifos | Organophosphate | 18.25 | 830 | (Sanchez-Bayo & Goka 2014) |
| Clothianidin | Neonicotinoid | 6.61 | 319 | (Sanchez-Bayo & Goka 2014; Bonmatin *et al.* 2015) |
| Coumaphos | Phosphorothioate | 105.5 | 5917 | (Sanchez-Bayo & Goka 2014) |
| Deltamethrin | Pyrethroid | 4 .6 | 91 | (Sanchez-Bayo & Goka 2014) |
| Fipronil | Phenylpyrazole | 33.6 | 70 | (Bonmatin *et al.* 2015) |
| Flumethrin | Pyrethroid | 6.7 | 158 | (Sanchez-Bayo & Goka 2014) |
| Flupyradifurone | Butenolide | 113.6 | 1800 | (Glaberman & White 2014) |
| Imidacloprid | Neonicotinoid | 8.43 | 912 | (Sanchez-Bayo & Goka 2014; Bonmatin *et al.* 2015) |
| Tau-fluvalinate | Pyrethroid (synthetic) | 15.9 | 2670.0 | (Sanchez-Bayo & Goka 2014) |
| Thiacloprid | Neonicotinoid | 41.86 | 187.6 | (Sanchez-Bayo & Goka 2014; Bonmatin *et al.* 2015) |
| Thiamethoxam | Neonicotinoid | 9.584 | 162.1 | (Sanchez-Bayo & Goka 2014; Bonmatin *et al.* 2015) |

**Table S3 The average and maximum concentrations of pesticide (ppb) residue found in the nectar content of flowers/crops and in nectar found in bee colonies. Field realistic acute exposure for both honey bees and bumblebees were worked out using the average amount of nectar ingested while a bee foraged. Values are based on Sanchez-Bayo & Goka (2014) and Bonmatin et al. (2015)**

| Pesticide | Pesticide Type | Average (ppb) | Maximum (ppb) | Honey bee average (ng/40mg) | Honey bee maximum (ng/40mg) | Bumble bee average (ng/37.7mg) | Bumble bee maximum (ng/37.7mg) |
| --- | --- | --- | --- | --- | --- | --- | --- |
| Acetamiprid | Neonicotinoid | 2.4 | 2.4 | 0.096 | 0.096 | 0.090 | 0.090 |
| Clothianidin | Neonicotinoid | 7.765 | 319 | 0.310 | 12.76 | 0.292 | 12.026 |
| Chlorpyrifos | Organophosphate | 3.9 | 15 | 0.156 | 0.6 | 0.147 | 0.565 |
| Coumaphos | Phosphorothioate | 105.5 | 2020 | 4.22 | 80.8 | 3.977 | 76.154 |
| Deltamethrin | Pyrethroid | 4.6 | 6.7 | 0.184 | 0.268 | 0.173 | 0.252 |
| Fipronil | Phenylpyrazole | 33.6 | 100 | 1.344 | 4.0 | 1.266 | 3.77 |
| Flumethrin | Pyrethroid | 6.7 | 158 | 0.268 | 6.32 | 0.252 | 5.956 |
| Flupyradifurone | Butenolide | 131.5 | 1500 | 5.26 | 60 | 4.95 | 56.55 |
| Imidacloprid | Neonicotinoid | 5.226 | 95.2 | 0.209 | 3.808 | 0.197 | 3.589 |
| Tau-fluvalinate | Pyrethroid (synthetic) | 15.9 | 750 | 0.636 | 30 | 0.599 | 28.275 |
| Thiacloprid | Neonicotinoid | 4.15 | 6.5 | 0.166 | 0.26 | 0.156 | 0.245 |
| Thiamethoxam | Neonicotinoid | 4.054 | 20 | 0.162 | 0.8 | 0.152 | 0.754 |

**Sensitivity analysis**

When the analysis was re-run using just data collected on honey bees, effects of pesticides on learning were significantly stronger for above field realistic dosages (p < 0.001) than for field realistic dosages (Figure S2). There were also significant difference between the effects of pesticide dosages on bee memory (p = 0.04) although both field realistic dosages still had an impact (Figure S2). There was no detectable difference between effects of chronic and acute exposure of pesticide on bee learning (p = 0.11) although there were differences in effects of chronic and acute exposure to pesticides on memory (p < 0.01) with chronic exposure resulting in stronger negative effects. We found no statistical differences in the learning score of (p = 0.09) or memory of (p = 0.47) *A. mellifera* and *A. cerana* and no differences between neonicotinoid pesticides and others (learning, p = 0.53; memory, p = 0.1). The results also showed no difference between short-term and long-term memory (p = 0.47).

We also re-ran the analysis excluding results that used a combination of pesticides (learning n = 2, memory n = 2). There were no significant differences in the learning data between bees that had been acutely and chronically exposed to pesticides (p = 0.73). There were significant differences between field realistic and above field realistic exposure (p < 0.01) with field realistic dosages having a stronger impact (field realistic, d = -0.16, 95% CL = -0.28 to -0.04; above field realistic, d = -0.36, 95% CL = - 0.47 to -0.25) and significant differences between *Apis* and *Bombus* (p < 0.05). The memory results did not change, as there were significant differences between pesticide effects on memory of acutely and chronically exposed bees (p < 0.05), significant differences between field realistic and non-field realistic exposure (p < 0.05), but not *Apis* and *Bombus* (p = 0.15), or short term and long-term memory (p = 0.67).


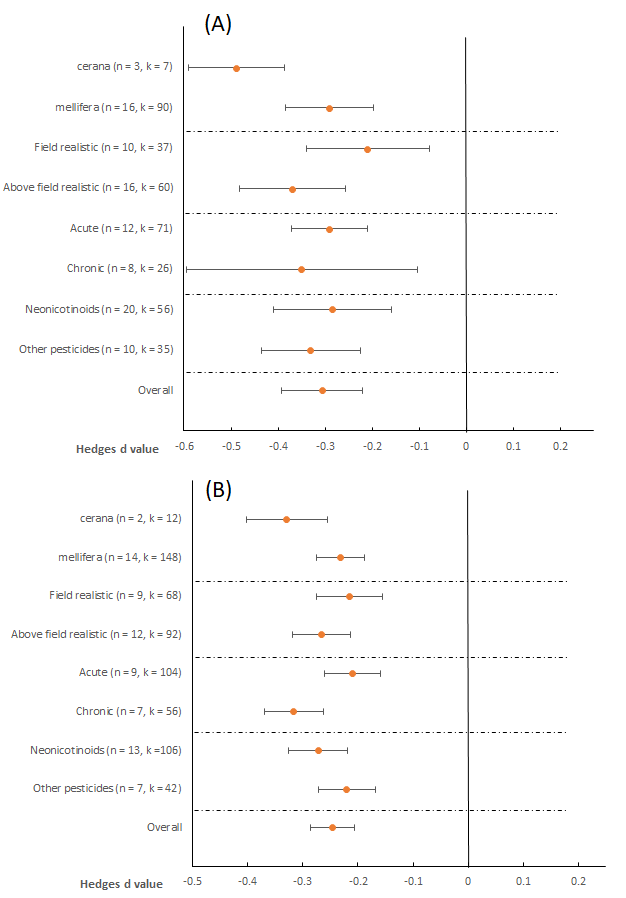


**Figure S2. Mean effect size estimates (± 95% confidence intervals), for subsets of the data when only the *Apis* data were analysed, for impacts of pesticides on (a) learning and (b) memory. Number of studies (n) and number of effect sizes (k) are given for each subgroup.**


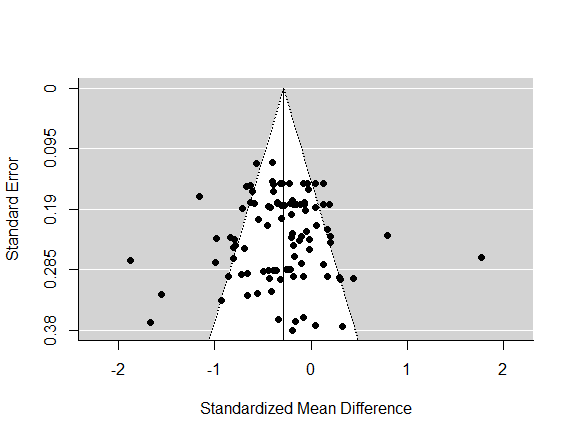
**Figure S3: Funnel plot for the learning data.**


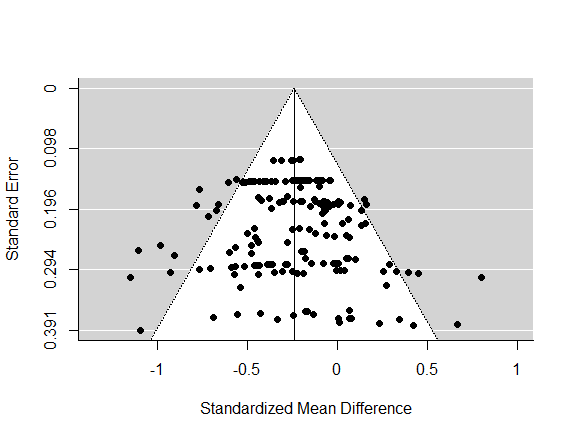


**Figure S4 Funnel plot for the memory data**

**References**

Abramson, C.I., Aquino, I.S., Ramalho, F.S. & Price, J.M. (1999) The effect of insecticides on learning in the africanized honey bee (*Apis mellifera L*). *Archives of Environmental Contamination and Toxicology*, **37**, 529–535.

Abramson, C.I., Sokolowski, M.B.C., Brown, E.A. & Pilard, S. (2012) The effect of pymetrozine (Plenum WG-50®) on proboscis extension conditioning in honey bees (*Apis mellifera*: Hybrid var. Buckfast). *Ecotoxicology and Environmental Safety*, **78**, 287–295.

Abramson, C.I., Squire, J., Sheridan, A. & Mulder, P.G. (2004) The effect of insecticides considered harmless to honey bees (*Apis mellifera*) proboscis conditioning studies by using the insect growth regulators tebufenozide and diflubenzuron. *Environmental Entomology*, **33**, 378–388.

Aliouane, Y., El Hassani, A.K., Gary, V., Armengaud, C., Lambin, M. & Gauthier, M. (2009) Subchronic exposure of honeybees to sublethal doses of pesticides: effects on behavior. *Environmental Toxicology and Chemistry*, **28**, 113–122.

Alkassab, A.T. & Kirchner, W.H. (2016) Impacts of chronic sublethal exposure to clothianidin on winter honeybees. *Ecotoxicology*, **25**, 1000–1010.

Bonmatin, J.M., Giorio, C., Girolami, V., Goulson, D., Kreutzweiser, D.P., Krupke, C., Liess, M., Long, E., Marzaro, M., Mitchell, E.A., Noome, D.A., Simon-Delso, N. & Tapparo, A. (2015) Environmental fate and exposure; neonicotinoids and fipronil. *Environmental Science and Pollution Research*, **22**, 35–67.

Bonnafe, E., Alayrangues, J., Hotier, L., Massou, I., Renom, A., Souesme, G., Marty, P., Allaoua, M., Treilhou, M. & Armengaud, C. (2017) Monoterpenoid-based preparations in beehives affect learning, memory, and gene expression in the bee brain. *Environmental Toxicology and Chemistry*, **36**, 337–345.

Burden, C.M., Elmore, C., Hladun, K.R., Trumble, J.T. & Smith, B.H. (2016) Acute exposure to selenium disrupts associative conditioning and long-term memory recall in honey bees (*Apis mellifera*). *Ecotoxicology and Environmental Safety*, **127**, 71–79.

Chakrabarti, P., Rana, S., Bandopadhyay, S., Naik, D.G., Sarkar, S. & Basu, P. (2015) Field populations of native Indian honey bees from pesticide intensive agricultural landscape show signs of impaired olfaction. *Scientific Reports*, **5**, 12504.

Ciarlo, T.J., Mullin, C.A., Frazier, J.L. & Schmehl, D.R. (2012) Learning impairment in honey bees caused by agricultural spray adjuvants. *PLoS ONE*, **7**, e40848.

Decourtye, A., Devillers, J., Cluzeau, S., Charreton, M. & Pham-Delègue, M.-H. (2004) Effects of imidacloprid and deltamethrin on associative learning in honeybees under semi-field and laboratory conditions. *Ecotoxicology and Environmental Safety*, **57**, 410–419.

Decourtye, A., Devillers, J., Genecque, E., Le Menach, K., Budzinski, H., Cluzeau, S. & Pham-Delègue, M.H. (2005) Comparative sublethal toxicity of nine pesticides on olfactory learning performances of the honeybee *Apis mellifera*. *Archives of Environmental Contamination and Toxicology*, **48**, 242–250.

Decourtye, A., Lacassie, E. & Pham-Delègue, M.-H. (2003) Learning performances of honeybees ( *Apis mellifera L*) are differentially affected by imidacloprid according to the season. *Pest Management Science*, **59**, 269–278.

Decourtye, A., Le Metayer, M., Pottiau, H., Tisseur, M., Odoux, J.F. & Pham-Delegue, M.H. (2001) Impairment of olfactory learning performances in the honey bee after long term ingestion of imidacloprid. *Hazards of Pesticides to Bees*, 113–117.

Frost, E.H., Shutler, D. & Hillier, N.K. (2013) Effects of fluvalinate on honey bee learning, memory, responsiveness to sucrose, and survival. *Journal of Experimental Biology*, **216**, 2931–2938.

Glaberman, S. & White, K. (2014) Environmental fate and ecological risk assessment for foliar, soil drench, and seed treatment uses of the new insecticide flupyradifurone (byi 02960). *U.S. Environmental Protection Agency Office of Pesticide Programs, Environmental Fate and Effects Division EFED, Environmental Risk Branch*, **187**, 56.

Goñalons, C.M. & Farina, W.M. (2015) Effects of sublethal doses of imidacloprid on young adult honeybee behaviour. *PLoS ONE*, **10**, e0140814.

Han, P., Niu, C.-Y., Lei, C.-L., Cui, J.-J. & Desneux, N. (2010) Use of an innovative T-tube maze assay and the proboscis extension response assay to assess sublethal effects of GM products and pesticides on learning capacity of the honey bee *Apis mellifera L*. *Ecotoxicology*, **19**, 1612–1619.

El Hassani, A.K., Dacher, M., Gary, V., Lambin, M., Gauthier, M. & Armengaud, C. (2008) Effects of sublethal doses of acetamiprid and thiamethoxam on the behavior of the honeybee (*Apis mellifera*). *Archives of Environmental Contamination and Toxicology*, **54**, 653–661.

Hesselbach, H. & Scheiner, R. (2018) Effects of the novel pesticide flupyradifurone (Sivanto) on honeybee taste and cognition. *Scientific Reports*, **8**, 4954.

Li, Z.G., Li, M., Huang, J.N., Ma, C.S., Xiao, L.C., Huang, Q., Zhao, Y.Z., Nie, H.Y. & Su, S.K. (2017) Effects of sublethal concentrations of chlorpyrifos on olfactory learning and memory performances in two bee species, *Apis mellifera* and *Apis cerana*. *Sociobiology*, **64**, 174–181.

Papach, A., Fortini, D., Grateau, S., Aupinel, P. & Richard, F.-J. (2017) Larval exposure to thiamethoxam and American foulbrood: effects on mortality and cognition in the honey bee *Apis mellifera*. *Journal of Apicultural Research*, **56**, 475–486.

Piiroinen, S., Botías, C., Nicholls, E. & Goulson, D. (2016) No effect of low-level chronic neonicotinoid exposure on bumblebee learning and fecundity. *PeerJ*, **4**, e1808.

Piiroinen, S. & Goulson, D. (2016) Chronic neonicotinoid pesticide exposure and parasite stress differentially affects learning in honeybees and bumblebees. *Proceedings of the Royal Society B: Biological Sciences*, **283**, 20160246.

Ramirez-Romero, R., Chaufaux, J. & Pham-Delegue, M.-H. (2005) Effects of Cry1Ab protoxin , deltamethrin and imidacloprid on the foraging activity and the learning performances of the honeybee *Apis mellifera* , a comparative approach. *Apidologie*, **36**, 601–611.

Ramirez-Romero, R., Desneux, N., Decourtye, A., Chaffiol, A. & Pham-Delegue, M.H. (2008) Does CrylAb protein affect learning performances of the honey bee *Apis mellifera L.* (Hymenoptera, Apidae)? *Ecotoxicology and environmental safety*, **70**, 327–333.

Rix, R.R. & Christopher Cutler, G. (2017) Acute exposure to worst-case concentrations of amitraz does not affect honey bee learning, short-term memory, or hemolymph cctopamine levels. *Journal of Economic Entomology*, **110**, 127–132.

Sanchez-Bayo, F. & Goka, K. (2014) Pesticide residues and bees – a risk assessment. *PLoS ONE*, **9**, e94482.

Schneider, S., Eisenhardt, D. & Rademacher, E. (2012) Sublethal effects of oxalic acid on *Apis mellifera* (*Hymenoptera: Apidae*): changes in behaviour and longevity. *Apidologie*, **43**, 218–225.

Stanley, D.A., Smith, K.E. & Raine, N.E. (2015) Bumblebee learning and memory is impaired by chronic exposure to a neonicotinoid pesticide. *Scientific Reports*, **5**, 16508.

Tan, K., Chen, W., Dong, S., Liu, X., Wang, Y. & Nieh, J.C. (2015) A neonicotinoid impairs olfactory learning in Asian honey bees (*Apis cerana*) exposed as larvae or as adults. *Scientific Reports*, **5**, 10989.

Tan, K., Wang, C., Dong, S., Li, X. & Nieh, J.C. (2017) The pesticide flupyradifurone impairs olfactory learning in Asian honey bees (*Apis cerana*) exposed as larvae or as adults. *Scientific Reports*, **7**.

Tan, K., Yang, S., Wang, Z. & Menzel, R. (2013) Effect of flumethrin on survival and olfactory learning in honeybees. *PLoS ONE*, **8**, e66295.

Thany, S., Bourdin, C., Graton, J., Laurent, A., Mathé-Allainmat, M., Lebreton, J. & Le Questel, J.-Y. (2015) Similar comparative low and high doses of deltamethrin and acetamiprid differently impair the retrieval of the proboscis extension reflex in the forager honey bee (*Apis mellifera*). *Insects*, **6**, 805–814.

Tison, L., Holtz, S., Adeoye, A., Kalkan, Ö., Irmisch, N.S., Lehmann, N. & Menzel, R. (2017) Effects of sublethal doses of thiacloprid and its formulation Calypso ® on the learning and memory performance of honey bees. *The Journal of Experimental Biology*, **220**, 3695–3705.

Urlacher, E., Monchanin, C., Rivière, C., Richard, F.-J., Lombardi, C., Michelsen-Heath, S., Hageman, K.J. & Mercer, A.R. (2016) Measurements of chlorpyrifos levels in forager bees and comparison with levels that disrupt honey bee odor-mediated learning under laboratory conditions. *Journal of Chemical Ecology*, **42**, 127–138.

Weick, J. & Thorn, R.S. (2002) Effects of acute sublethal exposure to coumaphos or diazinon on acquisition and discrimination of odor stimuli in the honey bee (Hymenoptera: Apidae). *J. Econ. Entomol*, **95**, 227–236.

Williamson, S.M., Baker, D.D. & Wright, G.A. (2013) Acute exposure to a sublethal dose of imidacloprid and coumaphos enhances olfactory learning and memory in the honeybee *Apis mellifera*. *Invertebrate Neuroscience*, **13**, 63–70.

Williamson, S.M. & Wright, G.A. (2013) Exposure to multiple cholinergic pesticides impairs olfactory learning and memory in honeybees. *Journal of Experimental Biology*, **216**, 1799–1807.

Wright, G.A., Softley, S. & Earnshaw, H. (2015) Low doses of neonicotinoid pesticides in food rewards impair short-term olfactory memory in foraging-age honeybees. *Scientific Reports*, **5**, 15322.

Yang, E.-C., Chang, H.-C., Wu, W.-Y. & Chen, Y.-W. (2012) Impaired olfactory associative behavior of honeybee workers due to contamination of imidacloprid in the larval stage. *PLoS ONE*, **7**, e49472.
